# Forward-Viewing Endoscopy at the Time of ERCP: A Blinded Tandem Prospective Trial

**DOI:** 10.1055/a-2932-0440

**Published:** 2026-08-21

**Authors:** Raj Shah, Kunal Jajoo, Steven N. Steinway, Thomas Wang, Thomas R. McCarty, Thomas J. Mathews, Roberto Trasolini, Pichamol Jirapinyo, Amit Grover, Linda S. Lee, Christopher C. Thompson, Marvin Ryou

**Affiliations:** 1 Division of Gastroenertology 12306 The Ohio State University Wexner Medical CenterColumbusOhioUnited States; 2 Division of Gastroenterology, Hepatology and Endoscopy 1861 Brigham and Women’s HospitalBostonMassachusettsUnited States; 3 Division of Gastrointestinal and Liver Diseases 5116 University of Southern CaliforniaLos AngelesCaliforniaUnited States; 4 Lynda K. and David M. Underwood Center for Digestive Disorders 23534 Houston Methodist HospitalHoustonTexasUnited States; 5 Internal Medicine 21638 The University of Kansas Medical CenterKansas CityKansasUnited States; 6 Department of Gastroenterology 12358 Faculty of Medicine, The University of British ColumbiaVancouverBritish ColumbiaCanada; 7 Division of Gastroenterology, Hepatology and Nutrition 1862 Boston Children’s HospitalBostonMassachusettsUnited States

**Keywords:** pancreatobiliary (ERCP/PTCD), diagnostic ERC, ERC topics, endoscopy upper GI tract, diagnosis and imaging (inc chromoendoscopy, NBI, iSCAN, FICE, CLE)

## Abstract

**Background and Study Aims** Currently, most endoscopists perform an ERCP with only a side-viewing endoscope. We hypothesized that given the non-forward viewing design of an ERCP endoscope, there is a significant miss rate of important findings. The aim of this study was to characterize the difference between a forward-viewing and a side-viewing exam during ERCP.

**Patients and Methods** We conducted a blinded, tandem, single tertiary center prospective clinical trial of patients undergoing ERCP. Patients underwent a standard forward-viewing endoscope exam with one attending. Findings were clinically significant, defined as leading to management change, or non-management altering. A blinded second attending subsequently performed an ERCP with a side-viewing endoscope.

**Results** In all, 163 participants were included. 69 (42.3%) patients had a finding that was found on forward-viewing exam, which was not found on side-viewing exam. Clinically significant findings, the primary outcome, were noted in 37 patients (22.7%) on forward-viewing endoscopic exam that were not seen on side-viewing exam. Comorbid malignancy was a statistically significant predictor of missed findings on side-viewing exam by univariate (30.4% vs 12.7%, *p* = 0.008) and multivariate ( *p* = 0.027) analyses. Surgically altered anatomy was also statistically significant on univariate analysis (<0.001).

**Conclusions** About 23% of the patients in this blinded, tandem prospective trial had a management-altering finding on forward-viewing endoscopic exam that was missed by a side-viewing endoscopic exam. These findings suggest that forward-viewing exam should be performed in conjunction with side-viewing exam during ERCP, particularly in the setting of malignancy or altered anatomy. ClinicalTrials.gov number, NCT05627882.

## Introduction

Endoscopic retrograde cholangiopancreatography (ERCP) plays an integral role in the work up and treatment of various pancreaticobiliary disorders. 1 2 Typically, the side-viewing duodenoscope is used for ERCP as it allows for better ampulla viewing and cannulation, while the forward-viewing endoscope is used for most other diagnostic and interventional needs in the upper gastrointestinal tract. 1 3 As its name implies, the side-viewing duodenoscope camera view is angulated and thus different than the forward-viewing esophagogastroduodenoscopy (EGD) endoscope. The duodenoscope has a field of view of 100 degrees, while the forward viewing gastroscope’s field of view is 140 degrees. 1

Although practice patterns vary, endoscopists traditionally use a side-viewing duodenoscope without a forward-viewing endoscope during an ERCP procedure. It is unclear if the view of the duodenoscope is sufficient for a thorough examination of the upper gastrointestinal tract during an ERCP procedure. The question of whether an (EGD is needed prior to ERCP has been proposed previously. One group highlighted that there is no evidence-based literature to recommend EGD prior to all ERCPs, but concluded that there was no indication to routinely perform an EGD prior to ERCP unless there were symptoms of dysphagia, clinical suspicion for a stricture or altered anatomy. 4 A retrospective study reported outcomes on patients who underwent both forward-viewing and non-forward viewing (ERCP or linear endoscopic ultrasound or both) endoscopy. It reported an 18% clinically significant miss rate by the non-forward viewing endoscope, but found on the forward-viewing exam. Specifically, in the duodenoscope cohort, 10 patients out of 52 (19.2%) found an additional lesion with the addition of a forward-viewing exam. 1

However, there is a lack of prospective data to guide endoscopists whether a forward-viewing exam should be done routinely during ERCP. The primary aim of this prospective study was to investigate missed findings and resulting practice changes with side-viewing endoscopic examination alone versus forward-viewing examination in conjunction to the side-viewing exam during ERCP.

## Patients/Material and Methods

### Study Design and Participants

We conducted a blinded, tandem, single tertiary center prospective clinical trial (NCT: NCT05627882) approved by the local Institutional Review Board. We adhered to the STROBE guidelines. 5 Adults (>18 years old) undergoing an ERCP were recruited to participate. At the time of procedure consent, patients were consented to participate in the study. Patients were also eligible for inclusion if undergoing more than one procedure such as endoscopic ultrasound (EUS) and ERCP as long as a conventional ERCP targeting ampulla cannulation was planned. Participants were excluded if they had undergone an EGD with ERCP previously as part of the study to avoid duplication of patient characteristics or if the indication required an EGD a priori. Demographics including age, gender, ethnicity, and inpatient versus outpatient procedure were collected. Anticoagulation use, altered anatomy, and presence of comorbidities including malignancy, cardiac, renal, and liver disease were also collected.

### Study Flow and Endoscopes

If the patient agreed to the study consent, they were enrolled in the study. Once the patient was sedated, the first endoscopist performed an upper endoscopy with a standard EGD endoscope (EG-760R, Fujifilm, Tokyo, Japan). The standard EGD endoscope has a field of view of 140°, distal end diameter of 9.2 mm, flexible diameter of 9.3 cm, instrument channel of 2.8 mm, and observation range of 2–100 mm. The degree of bending is as follows: up – 210°, down – 90°, and left and right – 100°. During the upper endoscopy, the first endoscopist would alert a nurse who was not part of the study of the upper endoscopy findings including location, if the findings were clinically significant or not, and if an intervention would need to be performed at the end of the ERCP. As the second endoscopist was outside of the procedure room, blinded to the results, he or she would be invited into the procedure room once the upper endoscopy was complete.

The second endoscopist would then perform an ERCP with the duodenoscope (ED-580XT, Fujifilm, Tokyo, Japan). The duodenoscope has a field of view of 100°, viewing angle of 5° retrograde, observation range of 4–60 mm, distal end of 13.1 mm, flexible portion of 11.3 mm, instrument channel diameter of 4.2 mm, and a distal detachable end cap. The degree of bending is as follows: up – 120°, down and left – 90°, and right – 110°. During the ERCP, the second endoscopist would call out the upper gastrointestinal findings. A nurse would document this so that answers could not be modified. At the end of the ERCP, the upper gastrointestinal findings were compared between the forward- and side-viewing exams and, if needed, an intervention such as biopsy or clipping was performed for routine patient care.

### Outcome Assessors and Assessment

The primary outcome was clinically significant different findings between the two endoscopic exams in each patient. Secondary outcomes included total differences, possibly clinically significant differences and non-significant differences. Moreover, we aimed to determine if baseline characteristics were risk factors for missing a lesion were present. The endoscopists performing the procedure were the outcome assessors. This included nine credentialed attending gastroenterologists. At the end of the ERCP, the two endoscopists involved with each procedure would review the nurse’s documentation and decide if their findings were different between endoscopes, which findings were clinically significant different (see definition under “Study definitions” later), and the location of the difference. Both endoscopists had to agree. If there were disagreements, this was resolved through discussion and would involve a third endoscopist if it could not be resolved through discussion. The final results were recorded in a secure HIPPA compliant Excel database. Follow up time for outcome determination was immediately post procedure.

### Study Definitions

A priori, several definitions were delineated to ensure study findings and demographics were consistent throughout the study. These definitions are summarized in Table 1 . A “difference” was defined by any endoscopic finding that was either possibly clinically significant or non-significant between the forward- and side-viewing exam. A “possibly clinically significant difference” (PCSD) meant that the finding was found on only forward-viewing or side-viewing endoscopic examinations and led to a management change such as an endoscopic intervention, recommendation change, or medication prescription. PCSD was then further categorized as “not clinically significant post pathology review” or “clinically significant difference” (CSD). The distinction between these two categories allowed for CSD to be truly management changing, while “not clinically significant post pathology review” included endoscopic findings that prompted a biopsy per endoscopist’s discretion, but pathology results revealed that it did not lead to an actual finding that dictated management changes. A “non-significant difference” (NSD) was a finding that was found only on forward-viewing or side-viewing endoscopic examination and not both, but did not lead to a management change. Anticoagulation use included patients who were actively on anticoagulation medications, but they could be held for or continued during the procedure. Malignancy was defined as an oncologic diagnosis eligible for systemic chemotherapy or surgical resection. Specifically, local excision and localized skin cancers were excluded from this definition. Patients with a history of malignancy, but who were cured were not counted in this category. Cardiac history included any cardiac-related issues including hypertension and hyperlipidemia. Given the high prevalence of steatosis and fatty liver reported in the literature and the under-reporting of this finding, we excluded this diagnosis as part of the definition of liver history. 6 7 Metastasis to the liver was included as liver history. Altered anatomy was defined as any surgical history of the esophagus, stomach or duodenum excluding percutaneous endoscopic gastrostomy tube placement.

**Table 1:** Study definitions.

Term	Definition
Difference	Any endoscopic finding found on only one exam: forward-viewing or side-viewing exam. This is an umbrella or overarching term as these were either possibly clinically significant or non-significant difference
Non-significant difference	A finding that was found only on forward-viewing or side-viewing endoscopic examination and not both, but did not lead to a management change
Possibly clinically significant difference	A finding that was found on only forward-viewing or side-viewing endoscopic examinations and initially led to a management change such as an endoscopic intervention, recommendation change, or medication prescription. This is an umbrella or overarching term that includes both “clinically significant difference” or “not clinically significant post pathology review”
Not clinically significant post pathology review	This was a possibly clinically significant difference as it led to a biopsy on only one type of exam, but after pathology review was deemed to not lead to management change
Clinically significant difference	A true management changing finding that was found on only forward-viewing or side-viewing exam, but not both and when applicable, confirmed to management changing after pathology review as well

### Statistical Analysis

Based on retrospective data, we assumed a 19% miss rate by a side-viewing exam compared to a forward-viewing exam. 1 With 80% power, 144 patients were required to detect statistical significance. Continuous data were compared using two-sample t-test or Wilcoxon rank-sum test and categorical data using Fisher’s exact test. Univariate and multivariable analyses were performed using logistic regression to determine significant predictors of missed findings (i.e., patient characteristics). Statistical significance was defined as a two-tailed P value <0.05. Statistical analysis was performed using SAS 9.4. This trial is registered NCT: NCT05627882.

## Results



**Video 1** An example of forward-viewing exam versus side-viewing exam can be seen in Video 1. This example highlights a missed diagnosis of Barrett’s esophagus with the side-viewing exam but found on the forward-viewing exam in a patient undergoing ERCP for choledocholithiasis without other comorbidities.


Between October 2022 and November 2023, 163 participants were recruited and included in the study. Common indications for ERCP included malignant biliary obstruction, choledocholithiasis, stent exchange, cholangitis, and jaundice. Emergent/urgent ERCPs were not part of exclusion criteria and were included as appropriate. No patients were excluded once they were consented and underwent the procedure. The median age was 63.3 years old with 62.6% of the included population being female. In all, 83 (50.9%) were outpatients. Baseline characteristics including co-morbidities of participants are shown in Table 2 . Based on the a-priori definitions, three patients with localized basal cell carcinoma and two patients with intraductal papillary mucinous neoplasm were not included under the malignancy category, resulting in 56.4% ( *n* = 92) of the population having malignancy. Common indications for ERCP included malignant biliary obstruction, choledocholithiasis, stent exchange, cholangitis, and jaundice.

**Fig. 1:**
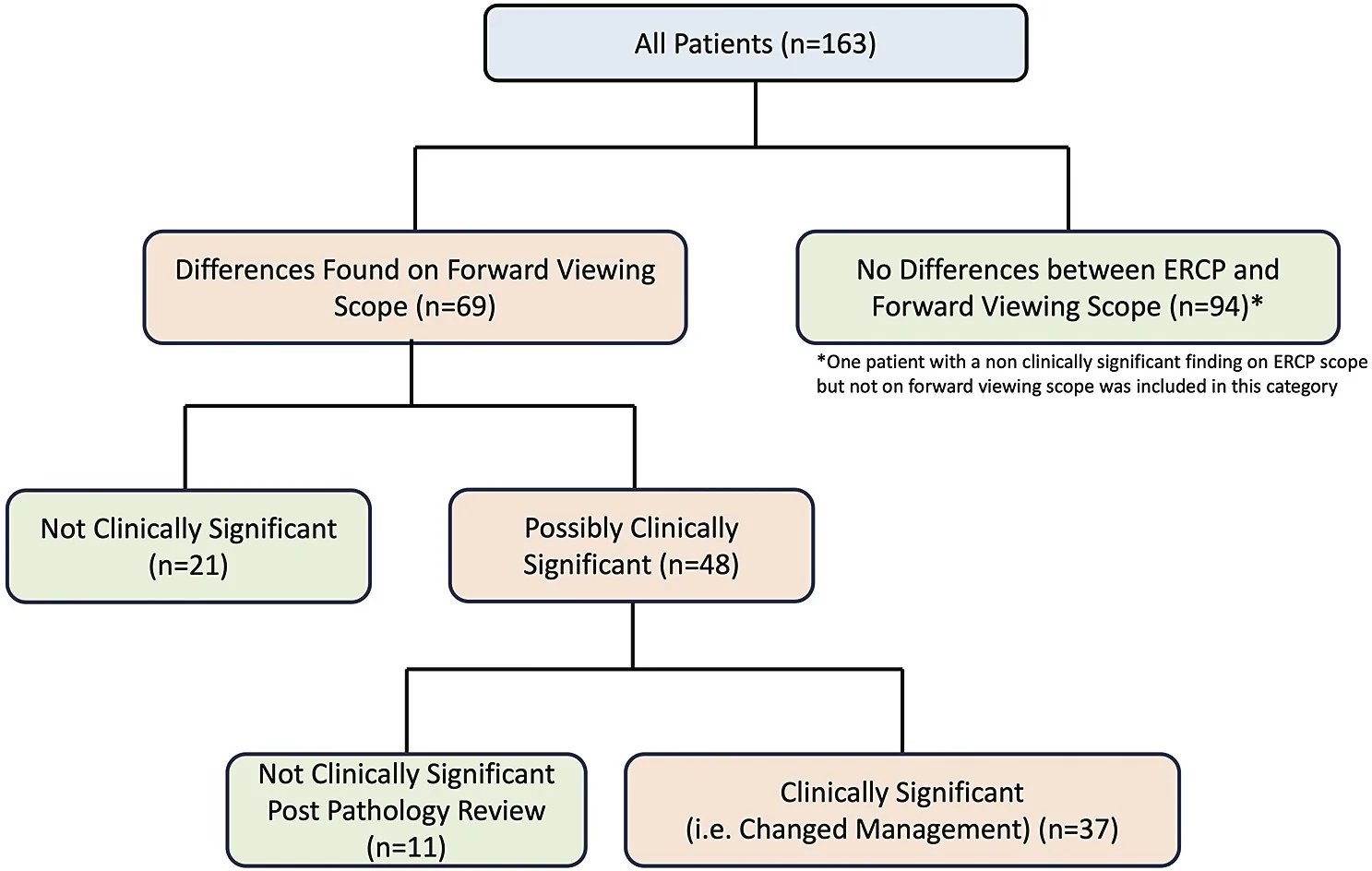
Results flow diagram.

The primary outcome, clinically significant difference, was found in 37 (22.7%) patients during forward-viewing exam that were missed on side-viewing exam. Management changes included: initiation or change in medication, change in endoscopic management or surveillance plan, need for anatomy delineation, or referral to a specialist ( Table 3 ). The “possibly clinically significant difference” cohort included 48 (29.4%) patients, with all findings found on forward-viewing exam and not on side-viewing exam. Distributions by location were as follows: 62.5% (30/48) in esophagus, 43.8% (21/48) in stomach, and 29.2% (14/48) in duodenum. List of these findings can be found in Table 4 . Totally, 6.7% (11/163) of the total population had a biopsy that did not lead to management altering plan due to the addition of a forward-viewing exam. One non-significant finding was found on side-viewing exam and not on forward-viewing exam. This was a clip in the peri-ampullary region. Results flow diagram can be found in Fig. 1 . Rates categorized by demographics and location for PCSD, NSD, and all differences can be found in Table 4 .

**Table 2:** Patient characteristics.

Patient Characteristics ( *n* = 163)	
Age (mean ± S.D.)	63.3 ± 15.3
Sex (female, *n* , %)	102 (62.6%)
Use of Anticoagulation	39 (23.9%)
Surgically altered anatomy	7 (4.3%)
Comorbidities	
Cardiovascular	90 (55.2%)
Renal	15 (9.2%)
Liver	29 (17.8%)
Malignancy	92 (56.4%)
- Pancreas cancer	35 (38.0%)
- Cholangiocarcinoma/liver	26 (28.2%)
- Breast	12 (13.0%)
- Colorectal	9 (9.8%)
- Genitourinary	9 (9.8%)
- Other	11 (12.0%)

**Table 3:** Type and frequency of possibly clinically significant findings on forward-viewing scope only (i.e., missed or not visible on side-viewing examination) ( *n* = 163).

Finding	# Times found	% Patients
LA Grade B esophagitis or higher	14	9.7
Esophageal varices	8	5.5
Duodenal ulcer	5	3.4
Duodenal stenosis	5	3.4
Altered surgical anatomy	5	3.4
Portal hypertensive gastropathy	4	2.8
Gastric polyp requiring resection or biopsy	3	2.1
Barret’s esophagus	3	2.1
Gastric ulcer	2	1.4
Gastric varices	2	1.4
Esophageal nodule with concern for malignancy	1	0.7
Gastric sarcoma	1	0.7
Large hiatal hernia	1	0.7
Duodenal erythema (history of Crohn’s disease)	1	0.7
How Clinically Significant Findings Changed Management ( *n* = 37)		
Initiated new or change in medication (e.g., PPI)	19	51.4
Changed endoscopic management/surveillance plan	10	27.0
Delineate altered anatomy to facilitate ERCP	7	18.9
New specialist referral	1	2.7

**Table 4:** Anatomic location and demographic data for possibly clinically significant differences, no significant differences, and all differences.

Parameter	Possibly clinically significant difference on forward viewing scope ( *n* = 48)	Clinically significant difference confirmed on pathology ( *n* = 37)	No significant differences on forward-viewing scope ( *n* = 21)	All differences ( *n* = 69)
Location, *n* (% of category total)				
Esophagus	30 (62.5%)	24 (64.9%)	7 (35%)	37 (53.6%)
Stomach	21 (43.8%)	12 (32.4%)	11 (55%)	32 (46.4%)
Duodenum	14 (29.2%)	11 (29.7%)	7 (30%)	21 (30.4%)
Clinical Characteristic				
Mean age (years)	63.9 ± 16.5	65.4 ± 15.0	68.9 ± 13.2	65.4 ± 15.6
Gender (F), *n* (% of category total)	25 (52.1%)	20 (54.1%)	15 (71.4%)	40 (58.0%)
Anticoagulation	15 (31.3%)	11 (29.7%)	5 (23.8%)	20 (29.0%)
Malignancy	36 (75.0%)	28 (75.7%)	12 (57.1%)	48 (69.6%)
Cardiac	27 (56.3%)	20 (54.1%)	11 (5.2%)	38 (55.1%)
Renal	6 (12.5%)	5 (13.5%)	3 (14.3%)	9 (18.8%)
Liver	12 (25.0%)	10 (27.0%)	4 (19%)	16 (33.3%)
Altered anatomy	7 (14.6%)	7 (18.9%)	0 (0%)	7 (10.1%)

Malignancy was a statistically significant predictor of missed finding on side-viewing exam by univariate (30.4 vs 12.7%, *p* = 0.008) and multivariate analyses ( *p* = 0.027). Surgically altered anatomy was also a statistically significant predictor of missed finding on side-viewing exam by univariate analysis (100 vs 19.2%, *p* = 0.001). The presence of cardiac, renal, liver comorbidities, anti-coagulation, and gender were not predictors of missed findings on side-viewing exam ( Table 5 ). No complications occurred due to the addition of a forward-viewing endoscope exam. An example of forward-viewing exam versus side-viewing exam can be seen in Video 1 . This example highlights a missed diagnosis of Barrett’s esophagus with the side-viewing exam but found on the forward-viewing exam in a patient undergoing ERCP for choledocholithiasis without other co-morbidities Video 1 .

**Table 5:** Univariate and multivariate analyses of patient characteristics between patients with clinically significant findings on forward-viewing scope only versus no difference in significant findings.

Characteristic	Patients with characteristic/comorbidity	Patients without characteristic/comorbidity	Univariate *P* -value	Multivariate *P* -value
Age (years)	65.4	62.7	0.34	0.30
Female sex	19.6%	27.9%	0.25	0.19
**Surgically altered anatomy**	100.0%	19.2%	<0.001*	N/A
Cardiovascular	22.2%	23.3%	1.0	0.26
Renal	33.3%	21.6%	0.33	0.46
Liver	34.5%	20.1%	0.14	0.20
**Malignancy**	30.4%	12.7%	0.008*	0.027*

**Note:** Seven demographics and comorbidities were evaluated for association with missed findings on forward-viewing examination: age, sex, anti-coagulation use, surgically altered anatomy, cardiovascular, renal, liver, and malignancy. Univariate and multivariate logistic regression were used to identify clinically significant patient features. Multivariate analysis was not performed for surgically altered anatomy given quasi-complete separation of data points secondary to 100% predictive value for clinically significant difference on forward-viewing scope. *Statistically significant result.

## Discussion

The rates of performing ERCP between 2002 and 2019 have remained stable. One study based on MarketScan Commercial Claims and Encounters and Medicare Supplemental database estimated 177,508 ERCP performed in 2019. 8 This suggests that ERCP will continue to be a major need in the diagnosis and treatment of pancreaticobiliary diseases, and thus optimizing current practice is needed. Despite the 2016 Thomas et al. study, many endoscopists do not routinely perform a forward-viewing exam with an EGD prior to ERCP. 1 4 Our blinded, tandem prospective trial found clinically significant findings were missed in 22.7% of patients who underwent side-viewing exam alone during ERCP compared to concomitant forward-viewing exam. The previous retrospective study by Thomas et al. found a 19.2% (10/52) clinically significant miss rate compared to our findings of 22.7%. Unlike the Thomas et al. study, our inclusion criteria did include patients with altered anatomy to further determine the rate among this population and assess if this is a predictive factor. Additionally, the Thomas et al. study was conducted in a retrospective manner and of a single endoscopist’s experience, while our study was performed in a blinded prospective tandem fashion, including nine endoscopists’ experience making it more applicable to practice. 1

In this study, both malignancy and surgically altered anatomy were individually statistically significant predictors of clinically significant missed findings that were found on EGD. This suggests these populations should undergo EGD prior to ERCP. In clinical practice for altered anatomy, the forward-viewing endoscope has been shown to be helpful in delineating anatomy to allow for correct limb intubation, locating the papilla and even used for cannulation in certain anatomy states. As endoscopists sometimes use a forward-viewing endoscope to delineate anatomy, this suggests that the field of view is not optimal in certain situations with a side-viewing duodenoscope alone and our study’s data bolsters this hypothesis. 9 In regards to malignancy, the true reason for why this is a high-risk population for clinically significant missed findings remains unknown. However, as malignancy often impacts the body in a systemic manner, we suspect this is a reason for additional findings found on exams such as ulcers, inflammation, stenosis and portal hypertension manifestations.

Although there is scant literature on EGD prior to ERCP, previous studies have shown benefit in performing EGD prior to endoscopic ultrasound when using an oblique-viewing echoendoscope. One study of 204 patients found a 15.7% miss rate of lesions that altered medical management and 9.8% of patients having a lesion found with EGD that impacted the subsequent endoscopic ultrasound exam. The mean time of performing an EGD was 5.5 min. Selected examples in this endoscopic ultrasound study of missed lesions included surgically altered anatomy, neoplastic lesions, GIST, Barrett esophagus, and hyperplastic gastric polyps. 10 This further supports the notion that in current practice a non-forward endoscope does not appear to be sufficient for a thorough upper gastrointestinal exam. Moreover, the esophagus mainly has been thought to be the only high-risk area for missed finding due to its tubular structure; however, our data show that the esophagus, stomach, and duodenum are all areas for clinically significant missed findings (24 esophagus, 12 stomach, 11 duodenum) given the angulation of a non-forward viewing endoscope. 1 Additionally, there was one missed non-clinically significant finding in the peri-ampullary region that was found on side-viewing exam and not on forward-viewing exam. This illustrates the need for both forward- and side-viewing fields of view for a complete upper gastrointestinal tract exam.

There are several limitations to this study. This study was a single-center study and not a multi-center study. However, nine different advanced endoscopists’ practices were assessed at a large tertiary care center increasing applicability. Although the knowledge of an ongoing study was present to the endoscopists, it was not possible to adjust for an endoscopist looking more carefully with a side-viewing exam or less carefully when knowing a forward-viewing exam would be performed. However, traditionally, a slow exam with torquing for multiple views is not performed during ERCP with a duodenoscope to attempt to assess the entire upper GI tract. Routine practice was encouraged and nine different endoscopist practice were assessed to minimize this potential risk of bias. We suspect that it is in an endoscopist’s interest to decrease scope changes for time and convenience and thus we hypothesize it is less likely that bias was instilled as a large number of significant clinical findings were still found. Additionally, the study was not a randomized controlled trial; however, it would be difficult to follow patients in the control group to assess if a difference was found clinically in the future. For example, significant esophagitis or Barrett’s esophagus may take many years to lead to another procedure and it would be unknown if this missed finding was present during the ERCP. Additionally, epidemiologic risk assessment such as recent upper endoscopy, helicobacter pylori infection, NSAID intake, alcohol and smoking status, family history of malignancy, gastrointestinal symptoms, and BMI may all play a potential role in determining who needs an EGD along with ERCP. As this was not studied, further research should be done to further identify high-risk populations, as EGD is not needed for all ERCPs.

However, with regard to strengths, the prospective, blinded tandem nature of this study with 163 patients adds significantly to the literature where there is a paucity of data. As the patients served as their own controls, data were collected prospectively and outcome assessors were blinded. The risk of bias is markedly decreased compared to a retrospective design where there is less control of these biases. 11

Several arguments have been made against adding an EGD to a side-viewing ERCP procedure. These include additional risk of adding an EGD, prolonged procedure and anesthesia time, and increasing healthcare cost. 4 This study aids to alleviate some of these concerns. No adverse events were seen with the addition of a forward-viewing exam. Although not formally studied as an outcome during this study, the average time for a diagnostic EGD has been reported as 5–10 minutes. 12 Future studies will be needed to assess cost-effectiveness; however, with a short procedure time, low risk profile, and a high missed management altering finding rate, EGD prior to ERCP appears to be warranted for certain patients.

Technological advancements may also minimize barriers to implementation. The need for enhancing the field of view or adding a forward-viewing option to traditional angulated endoscopes has been studied before. For example, forward-viewing linear endoscopic ultrasound has been studied and been shown to be feasible for sampling and assessing subepithelial, hilar, proximal colon, and pancreatic lesions. 13 14 15 16 17 18 19 20 21 22 23 In the current state, there are limitations given scope design including difficulty accessing lesions from the second portion of the duodenum. 15 However, feasibility has also been shown using a forward-viewing linear EUS to perform interventions such as pancreatic fluid collection drainage, biliary drainage, gastric varices treatment, and celiac plexus neurolysis. 15 17 19 21 24 25 26 27 28 29 In regard to expanding the field of view, the full spectrum endoscope (FUSE®, EndoChoice Inc., Atlanta, Georgia, USA/Boston Scientific, Marlborough MA) was developed and allows for 245° view for gastroscope and 330° for colonoscope as there are imaging capabilities on the side of the scope as well as the forward tip. These endoscopes have been shown to likely enhance colon adenoma detection and aid with surveillance in patients with familial adenoma polyposis given the ability to assess the ampulla. 30 31 Once optimal endoscopes that can increase our field of view, are user-friendly, are cost-effective, and increase our ability to provide interventions are developed, their adoption is likely inevitable. The advancement of a single endoscope that could function in both a side-viewing and forward-viewing fashion may minimize missed findings and circumvent the current barriers to implementation of adding an EGD to ERCP. At the current time, the advanced endoscopy community should consider the addition of a forward-viewing exam with an EGD during ERCP.

## Conclusion

In the current practice, these results show that there is nearly a 23% clinically significant miss rate in patients undergoing ERCP with only a side-viewing endoscope, especially in patients with history of malignancy and surgically altered anatomy. These findings suggest that a concomitant forward-viewing exam with an EGD should be added during ERCP. Future studies will need to assess if there are additional predictors for missed findings, cost-effectiveness, interventions to minimize barriers to implementation of the addition of an EGD, and how practice changes based on the results of this study. Technologic advances minimizing missed findings may decrease the need for the addition of an EGD. However, currently adding a forward-viewing exam to the traditional side-viewing exam during ERCP is likely to decrease clinically significant missed findings.
